# Pharmacists' perceptions of pharmacy technician occupational values

**DOI:** 10.1016/j.rcsop.2023.100358

**Published:** 2023-11-04

**Authors:** Wesley Sparkmon, Marie Barnard, Meagen Rosenthal, Shane Desselle, Jordan Marie Ballou, Kristin L. Cullen-Lester, Erin R. Holmes

**Affiliations:** a Creighton University School of Pharmacy and Health Professions, 2500 California Plaza, Omaha, NE 68178, United States; b University of Mississippi School of Pharmacy, PO Box 1848 University, MS 38677, United States; c Touro University California College of Pharmacy, 1310 Club Dr Vallejo, CA 94592, United States; d University of South Carolina College of Pharmacy, 715 Sumter St Columbia, SC 29208, United States; e University of Mississippi School of Business Administration, PO Box 1848 University, MS 38677, United States

## Abstract

**Background:**

Pharmacy technician scope of practice has expanded in recent years to attempt to alleviate the responsibility burden placed on pharmacists in some states. However, little research has examined the ways in which pharmacists attempt to persuade technicians to take on additional roles. Management literature has identified the importance of understanding employee values in crafting persuasive role expansion messaging. Objectives: Identify the occupational values which pharmacists believe are the most important to pharmacy technicians when attempting to craft messages aimed at increasing technician involvement in advanced roles.

**Methods:**

Semi-structured interviews were conducted with pharmacists across multiple practice settings to identify how important they believe nine selected occupational values are to pharmacy technicians. Average scores for each of the nine values were calculated and examined to identify potential differences between the two overarching types of occupational values: intrinsic and extrinsic.

**Results:**

Pharmacists indicated that they believed that technicians are more extrinsically motivated than intrinsically motivated. Pharmacists believed that technicians had higher levels of extrinsic occupational values as opposed to intrinsic occupational values (3.920 vs. 3.113). The most important values to technicians as perceived by pharmacists were the income of the job and the hours of the jobs (average score of 4.85 and 4.75, respectively). The chance to be helpful to others and society was the only intrinsic value with an average score >3.5. Additionally, pharmacists indicated that technicians were not properly compensated for their work, which furthered illustrated the perceived importance of extrinsic motivators. Finally, when it came to crafting messaging around role expansion, pharmacists believed it was important to tailor their messaging to the technician they were speaking to.

**Conclusion:**

Pharmacists looking to craft role expansion messaging to their technicians are more likely to utilize extrinsic occupational values as motivators instead of using intrinsic values.

## 1 Introduction

Research on pharmacy technicians' role expansion has been primarily driven by examining the tasks pharmacy technicians can perform, how well they perform those tasks, and the effects of their role expansion on the pharmacy. Researchers have also focused on the recruitment of pharmacies in implementing an expanded technician role and the impact of technician role expansion. While this research is pertinent to the practice of pharmacy, little attention has been given to how the values of technicians impact their likelihood of taking on these additional roles. In particular, there has been minimal discussion of the methods researchers and pharmacists use to convince technicians to take on additional roles.

Researchers who have investigated why pharmacy technicians decide to take on expanded roles in the pharmacy have reported on certain variables that have contributed to technician involvement in specific tasks.^1^^,^^2^ In particular, these studies have identified intrinsic and extrinsic factors and provided some insight into their effect on technicians and additional roles. However, the occupational values describing these overarching factors that can increase the likelihood of technician role expansion in certain situations have not been thoroughly identified and investigated.

### 1.1 Occupational values and roles

The effect of employee values on employee attraction, commitment, and motivation in the business and service industries has long been studied in the jobs literature.^3^ One framework for studying the effect of values drew on the societal and sociological literature to posit that individuals who placed greater importance on financial rewards were more likely to pursue employment within the business sector, while individuals with more altruistic tendencies who valued service to others were more likely to enter into service professions.^4^ Researchers later realized that although values can justify job activities within job design, the nature of values meant that investment into the meaning of a job did not come through the organization but was a consequence of the employee's occupational values.^5^

Regarding these justifications by employees, studies of occupational choice behavior among undergraduate students consistently showed that people who place high importance on philanthropic values were more likely to enter the service industry, while students who placed importance on pecuniary outcomes were more aligned with a career in business.^6^ The studied values would be categorized into two clusters; intrinsic motivation and extrinsic motivation.^4^^,^^6^^,^^7^ However, separating the types of industries into simply business and service omitted industries that contained elements of both within its normal practice. One such industry was the practice of pharmacy. Studies showed that those in the practice of pharmacy were seen to have high levels of importance in both philanthropic values and financial outcomes.^4^

### 1.2 Occupational values and role expansion

One of the biggest issues facing organizations is differences in role definitions among individuals with the same job title and description.^8^^,^^9^ Vagueness in role definition can create uneven performance within the organization, with some employees going above and beyond their duties and some falling well short of expectations. Employees who define their roles more broadly, to include more tasks as part of their regular responsibilities, are more motivated to perform their tasks and perform those tasks more proficiently and proactively.^10^ There is evidence that role definitions are, in part, impacted by differences in values.^8^ Management research suggests that organizations may encourage broader role definitions (i.e., individuals taking on more responsibilities in the organization) by understanding employees' values.

Studies have indicated that more than three-fourths of all role expansion situations in an organization involve direct requests from one individual to another,^8^^,^^11^, ^12^, ^13^ supporting the traditional role theory perspective of role expansion. This perspective argues that role expansion decisions traditionally involve messaging, where a sender presents a new role to a receiver.^14^ This illustrates the importance of the interpersonal dynamics between pharmacists and technicians in understanding pharmacy technicians' role expansion, as this interaction (or social exchange within the theoretical framework) provides an example of persuasion by the pharmacist.^15^ Because of the persuasive nature of role expansion, the sender (e.g., a pharmacist) needs to understand what values are important to the receiver (e.g., a pharmacy technician). The expectancy theory of work motivation specifies that an individual is more likely to commit their time, energy, and attention to a situation when they believe that the situation will fulfill their values.^16^ Research has supported the expectancy theory, whereby individuals recruited to volunteer for a project were more persuaded by messaging that matched their values.^17^ Senders often struggle to understand the values of receivers, making research aimed at understanding pharmacy technicians' values of vital importance.

Literature in social psychology has shown a human tendency to assume that the values of other individuals are aligned with our own, leading senders to frame role expansion opportunities within the values they believe the receiver has instead of tailoring messaging to the receiver's actual values.^18^ Because of the potential effect on messaging, understanding pharmacists' perceptions of technicians' occupational values and how that perception shapes messaging could provide tremendous insight into how likely technicians are to take on additional roles in the pharmacy.

Initial evidence of likely misunderstandings between pharmacists and technicians is provided by an editorial describing responses to questions about the appropriateness of journal articles for technicians.^19^ The authors show a disconnect in understanding between pharmacists and technicians from a pharmacist's perspective. In one study, all surveyed pharmacy directors believed that journal articles were too complex for technicians to comprehend, while responses from technicians showed that just over 5% of technicians found the articles too complex, and nearly 90% found them appropriate for their level of understanding.^19^

Grant and Hofmann (2011) have provided a theoretical framework that illustrates the importance of messaging on role expansion and how occupational values and the alignment of those values can impact the likelihood of role expansion.^8^ However, it is currently hard to apply this framework to pharmacy technicians because there is minimal research into the occupational values of technicians and which values pharmacists believe to be important to technicians. To address this, we have developed an exploratory study to gain additional understanding of the occupational values pharmacists believe pharmacy technicians consider important regarding role expansion and how those perceived values impact how the pharmacist shapes their messaging to the pharmacy technician when approaching them about role expansion.

## 2 Methods

Interviews were conducted to explore what occupational values pharmacists believe are important to pharmacy technicians. Semi-structured interviews were employed with ten hospital pharmacists and ten community pharmacists to obtain more thorough information from pharmacists. The consolidated criteria for reporting qualitative research (COREQ) was utilized to conduct and analyze this study.^20^ The lead author (male) conducted these interviews as part of his doctoral dissertation in pharmacy administration He had course work and prior research experience utilizing qualitative research methods. This study was deemed exempt by the Institutional Review Board of the University of Mississippi.

The importance of occupational values on motivation is central to this study. In efforts to craft those messages, the research team aimed to measure how pharmacists believed technicians ranked the importance of nine occupational values. During the interviews, pharmacists were asked to rank nine values for how important they believe those occupational values are to pharmacy technicians when asking the pharmacy technician to take on an additional role. The nine values, adapted from Kronus, are the income of the job, the prestige of the career, the chance to exercise leadership, the chance to be helpful to others and to society, the chance to work with people rather than things, the chance to be original and creative in their work, the ability to live and work in the world of ideas, the hours and security of the job, and increased autonomy within their work.^4^ Within those values, five values (income, prestige, leadership, hours, and autonomy) are classified as extrinsic values, with the other four (helpful to society, working with people, original and creative, and working with ideas) are considered intrinsic.^4^^,^^6^

In these interviews, pharmacists were asked to provide role expansion messages for technicians based on what values they believe are important to technicians. Roles that were used for constructing messages are from another study completed as part of this project, in which a group of pharmacists were asked about which additional tasks they believed technicians should have an increased role in performing.^21^ Using these additional roles, pharmacists were asked to create messaging to persuade technicians to take on the additional role shaped by their perception of technician values and how they believe the message should be conveyed. The interview guide that was utilized during this phase of exploratory research is provided in Appendix A.

### 2.1 Sample

For this study, in-depth interviews were conducted with ten community pharmacists and ten hospital pharmacists. Pharmacists were purposively sampled and recruited via email to gather information from different pharmacy settings. Recruited pharmacists were required to have direct contact with their technicians and from multiple states with different requirements and permitted tasks among technicians. Purposive sampling was used for both community and hospital pharmacists to account for differences in tasks as part of normal scope of practice in each setting. Interviews were conducted either telephonically or using Zoom video conferencing software and recorded to ensure accurate transcription. Each interview took ten to fifteen minutes to complete.

### 2.2 Analysis

Interviews were transcribed using transcription software, then corrected by three pharmacy students. Transcripts were then analyzed by two researchers who independently coded interview responses using the nine occupational values adapted from Kronus to obtain numeric values for each value.^4^ After independent coding, the two researchers compared and triangulated their coding results.^22^ Steps were taken by researchers to establish the validity and reliability of responses and coding. By having multiple researchers code responses and a secondary opportunity for researchers to check responses and values from interviews, the face validity of responses is enhanced, and the reliability of responses was demonstrated.^23^

After calculating average values among intrinsic and extrinsic values, a paired *t*-test was conducted to test for any statistically significant difference between the perceived importance of intrinsic values and extrinsic values.

## 3 Results

Multiple topics were identified through the interviews conducted as a part of this study. In particular, pharmacists illustrated three key themes. First, pharmacists believe technicians are more motivated by extrinsic than intrinsic values. Secondly, pharmacists believe that technicians need to be more properly compensated for their work. And finally, pharmacists need to understand their technicians when attempting to persuade technicians to take on additional roles within the pharmacy.

### 3.1 Demographics of responding sample

In total, 20 pharmacists were interviewed as part of this study. Twelve of the 20 interviewed pharmacists (60%) listed their primary practice site as a community pharmacy, either open-door or closed-door. Eleven respondents were female (55%), and 18 of 20 respondents (90%) had received their Pharm.D. Eight of 20 interviewed pharmacists (40%) had also completed a post-graduate residency. A full description of the responding pharmacist sample is shown in Table 1.

**Table 1:** Demographics of Interviewed Pharmacists.

Demographic Trait		n (%)
Sex		
	Female	11 (55%)
	Male	9 (45%)
Education		
	Pharm.D.	18 (90%)
	B.S. Pharmacy	2 (10%)
Post-Graduate Residency		
	Yes	8 (40%)
	No	12 (60%)
Primary Practice Site		
	Community Chain	10 (50%)
	Hospital	8 (40%)
	Closed-Door Community	2 (10%)
Primary State of Practice		
	Mississippi	9 (45%)
	Kentucky	5 (25%)
	Tennessee	3 (15%)
	Indiana	1 (5%)
	Minnesota	1 (5%)
	Missouri	1 (5%)

### 3.2 Themes identified from interviews

#### 3.2.1 Theme 1: technicians are more motivated by their extrinsic values

The mean response scores indicate pharmacists believe technicians are more motivated by extrinsic than intrinsic values (Table 2). There was a statistically significant difference between pharmacist perceptions of the importance of extrinsic and intrinsic values to technicians (*p* < 0.005).

**Table 2:** Pharmacist Perceptions of Technician Occupational Values.

Values	N	Minimum	Maximum	Mean	Std. Dev.
Ex 1 – Income	20	4	5	4.85	0.366
Ex 2 – Prestige	20	2	4	3.1	0.788
Ex 3 – Leadership	20	1	5	2.95	0.887
In 1 – Helpful	20	2	5	3.95	0.759
In 2 – People	20	2	4	3.1	0.788
In 3 – Original	20	1	5	2.8	1.24
In 4 – Ideas	20	1	4	2.6	0.995
Ex 4 – Hours	20	3	5	4.75	0.55
Ex 5 – Autonomy	20	3	5	3.95	0.686
Extrinsic Average	20			3.920	0.369
Intrinsic Average	20			3.113	0.631

The importance of extrinsic values to technicians, as perceived by pharmacists, was made clear in some comments from interviewed pharmacists. One community pharmacist echoed the emphasis on extrinsic motivation, saying, *“this may not be the end road for [the technician]. [It] would be a good opportunity to show the technician's organizational skills, show leadership skills, and kind of practice those out in a real work setting.”* A hospital pharmacist also noted of extrinsic motivation:

*“I know there's a pull right now to get our [transitions of care] techs to have an increase in pay. I know that there's been overhearing them like ‘we could go do less work in a more specialty position…and get paid twice as much. (In regard to lower scores for original and creative values) I ranked [that value] a little bit lower because unfortunately there's only so much that you can do when you're taking a med history and there's standardized processes across [our hospital].”*

Part of this quote illustrates the perceived importance of one particular value according to pharmacists. Pharmacists believe that the income of the job is the most important to technicians when it comes to technicians taking on an additional role.

#### 3.2.2 Theme 2: technician salaries are not commensurate with their workload

As seen in Table 2, pharmacists believe that the greatest motivator of technicians is the income of the job, with a mean score of 4.85, a standard deviation of 0.366, and the minimum importance being 4. Pharmacists frequently mentioned an increase in salary as one of, if not their first, talking points when asked to craft task expansion messages to technicians.

One community pharmacist lamented that *“It has been so hard to find good technicians that want to stay, that don't get offered a job somewhere else with a higher pay, especially in the community pharmacy world.”* Another community pharmacist added, *“I'm going from store to store. It's just across the board, they [technicians] believe they're overworked and underpaid, and I see that as well. They do so much from the minute they walk in the door to the minute they leave.”*

Hospital pharmacists had the same sentiment as their community counterparts, with one stating, *“One thing that we're pushing [at our hospital] and [pharmacists] are all supporting is an increasing in pay grade, because if you take on that much more, it is a lot.”* Another hospital pharmacist said, *“I think that [technician motivation] is more money driven. They really just don't get paid enough.”* These sentiments were shared by almost all interviewed pharmacists, many of whom derided low pay as a reason for technician turnover and difficulty in hiring new technicians.

#### 3.2.3 Theme 3: pharmacists need to tailor role expansion messaging to technicians

The final theme which was identified was the necessity to tailor messaging to the specific technician when attempting to persuade the technician to take on additional roles. Within this study, researchers aimed to elicit messages from pharmacists that corresponded to the occupational values they believe are important to technicians. In framing these messages, many pharmacists noted the importance of tailoring messages to specific technicians. Some pharmacists emphasized the importance of having *“some kind of positive relationship with the technician”* and *“knowing your audience”* when communicating role expansion opportunities. Multiple pharmacists differentiated their approaches with statements like *“So if I had a technician who is motivated from the idea that they were getting to work with people and instead of things”* or *“From my experience, knowing your staff your technicians and getting a sense of those that really do want to.”* Even within the same value, some pharmacists argued that a single value, such as compensation, may have messaging tailored to a specific element of that value. As one pharmacist said *“it's important to make sure you're compensated in some way. Whether that's through, you know, a pay raise, or more flexible hours, or more vacation time.”* Tailoring a message from a pharmacist to a technician provides multiple opportunities for specifying the benefits that will best motivate a technician to take on an advanced role.

## 4 Discussion

This study is one of the first to qualitatively assess pharmacists' perceptions of why technicians might be motivated to take on additional roles within the pharmacy and the occupational values of pharmacy technicians. Previous studies of occupational values in the pharmacy setting have only focused on the occupational values of pharmacists and their effect on pharmacists' roles.^4^ This study provides a baseline knowledge of technician values as perceived by pharmacists, who directly oversee multiple technicians in their normal practice.

The theme developed in this paper is the pharmacists' perceptions of what occupational values are most important to technicians in terms of motivating technicians to take on an expanded role. Interviews illustrated that pharmacists across multiple settings believed that technician motivation was most highly driven by extrinsic motivators, particularly the job's income and the job's hours and security. Some of that belief could be driven by the atmosphere in pharmacies at the time of the interviews. International studies show burnout among more than half of surveyed technicians coming out of the COVID-19 pandemic.^24^ Lay media have reported issues with technician burnout due to dealing with frustrated customers, increased focus on metrics by employers, and an increased workload without additional staff to assist with the increased burden.^25^

In this study, multiple community pharmacists specifically cited working with people rather than things as something they believed could be less of a motivator than it once was. The ability to work with people rather than things and the chance to be helpful to others and society both had lower average scores among community pharmacists than hospital pharmacists (2.92 vs. 3.38 and 3.83 vs. 4.13, respectively). Some pharmacists even laughed, saying only *“Retail”* when asked about the importance of working with people rather than things to technicians, illustrating the current issues which are felt by pharmacy staff in the community retail setting. This provides another opportunity for future research, particularly coming out of the COVID-19 pandemic, examining burnout among technicians and the reasons that they may cite.

The second identified theme was that the income of the job was perceived as the most important value to technicians by nearly all pharmacists, but many of those same pharmacists specifically cited that technicians were not adequately compensated for their work. Recent studies by the Bureau of Labor Statistics (BLS) found the average hourly wage for a nationally certified pharmacy technician (CPhT) was between $14 and $19 an hour depending on the level of experience of the technician, with average annual salaries of less than $40,000 a year.^26^ Studies looking at BLS data of pharmacist and technician salaries over a 20-year period noted a lower growth in technician salaries despite their additional responsibilities.^27^ Previous studies have had pharmacists who expressed opinions that pharmacy technicians were already not paid enough, so it would be unfair to expect technicians to take on additional responsibilities without a pay increase.^28^ A similar attitude was held by pharmacists in this study, whereby multiple pharmacists discussed efforts within their organizations to increase technician salary. Repeated calls for increased technician wages could shape future studies into technician motivation.

The final theme from these interviews was the need for pharmacists to tailor role expansion messaging to the specific technician. Responses from pharmacists often included caveats before providing messages where they noted they would mold their messages to most appropriately fit what they believed was important to their particular technician. Many interviewed pharmacists discussed the importance of adapting messaging to their employees, but they may not realize the role occupational values might play in persuading a technician to take on additional roles within their pharmacy. By better understanding what technicians value, pharmacists may become more knowledgeable on how to tailor their messages moving forward.

The argument for tailoring messaging supports the theoretical framework of this study, wherein management theorists have often cited the importance of dyadic communication in role expansion between managers (i.e., pharmacists) and employees (i.e., technicians).^8^^,^^14^^,^^15^ This social exchange process where the sender attempts to convince the receiver to take on an additional role as part of their normal job responsibilities is theoretically vital to the employee taking on the new task as part of their work. Though they may not be aware, pharmacists arguing the importance of message tailoring are arguing based on the expectancy theory of work motivation, where individuals are most likely to devote their time and energy to opportunities that they believe will permit them to express or fulfill their values.^16^ Based on what pharmacists believed was important to technicians, pharmacists tailored their messaging in response to what they believed was important to technicians. This message tailoring helped to contribute to our larger project examining this relationship between pharmacists' messaging, technicians' value, and the likelihood for technicians to take on additional responsibilities within their work.

The potential difference in what pharmacists perceive as important to technicians as opposed to what is actually important to technicians provides a potential avenue for research examining where differences exist between the two populations and why those differences may exist. This is not the only area that merits further examination by researchers, as many of the repeated themes within the interviews provide interesting opportunities to grow understanding about and among pharmacists and technicians.

As with all qualitative interview studies, there are inherent biases and limitations in the responses given by individuals. Responses can provide great insight into the values of technicians those pharmacists work with, but those responses do not provide information regarding all technicians. Responses are also not generalizable to the beliefs of pharmacists as a whole. Additionally, the semi-structured nature of the interview presents potential issues with analysis and different depths of responses. Also, the sample size of the study limits the generalizability. This study was completed as part of a larger project completed as part of a dissertation project, which limited time and incentive resources for a larger sample. The findings of this study provide a baseline understanding which can be tested among a larger sample of pharmacists in the future. However, despite their limitations, semi-structured interviews offer researchers a great deal of expanded knowledge.

## 5 Conclusion

The relationship between a pharmacist and a technician is vital to the success of a pharmacy, as many pharmacists stated that nothing in the pharmacy could happen without the assistance of a technician. The understanding built in this relationship can help pharmacists to persuade technicians to take on additional tasks within the technician's normal scope of practice. This study examined which occupational values pharmacists believe are important to pharmacy technicians regarding the expansion of technician scope of practice and how those perceived important values impact the crafting of persuasive messaging by pharmacists for pharmacy technicians. Given the framework of role theory upon which this study was designed, it is important to understand what factors may impact how the message sender presents the new opportunity. This work, combined with Vroom's expectancy theory of work motivation,^18^ positing that message receivers are more likely to take on responsibilities that fulfill their values, provides a foundation to build further studies on the role of occupational values and their impact on the practice of pharmacy moving forward.

## Declaration of Competing Interest

None.

## Appendix A Supplementary data

Supplementary material: Interview Guide Image 1
