# Supplementary material for: Pharmacists' perceptions of pharmacy technician occupational values
Source: Explor Res Clin Soc Pharm. 2023 Nov 4;12:100358. doi: 10.1016/j.rcsop.2023.100358 (PMC10685301; doi:10.1016/j.rcsop.2023.100358)
Supplement: Supplementary file 1 — Supplementary material: Interview Guide [file mmc1.docx]

**APPENDIX A – Interview Guide**

Thank you for your participation in this study. We just have a few questions today to ask. This interview should take approximately 20-30 minutes. Your responses will be recorded anonymously and any statements you make will be deidentified.

To begin, I just want to ask if I have consent to record this interview to make sure that we don’t miss anything?

Are you 18 years of age or older?

**PHARMACY DEMOGRAPHICS**
How would you describe your primary pharmacy practice site?

What is your primary site of practice?

Approximately how many prescriptions does your pharmacy fill per day?

Approximately how many technicians work with you on a normal day?

Are all of your technicians required to be nationally certified? Is that board of pharmacy policy or organizational policy?

(If not all technicians are certified) Do you encourage your technicians to pursue national certification?

**TECHNICIAN SCOPE OF PRACTICE**
What tasks do you expect your technician to complete within their normal scope of practice?

Do you think your state’s rules and regulations surrounding pharmacy are too relaxed, too stringent, or about right when it comes to technician scope of practice? Why?

Are there any tasks which you believe your technicians could/should assist with which they are not already?

**COVID QUESTIONS**

Did the COVID-19 Pandemic lead to any changes in tasks your technicians performed in your pharmacy? What were the changes?

Yes/No

Do you believe that technician scope of practice should expand after the end COVID-19 public health emergency which has expanded technician scope of practice to include administering immunizations?

Yes/No

Did your opinions of technician scope of practice change after the COVID-19 pandemic? Why?

Yes/No

**TECHNICIAN VALUES**
On a scale of 1-5, how do you rank the following values do you believe are important when it comes to why a technician would take on additional roles within the pharmacy?

1. The Income of the Job
2. The Prestige of the Career
3. The Chance to Exercise Leadership
4. The Chance to be Helpful to Others and to Society
5. The Chance to Work with People Rather than Things
6. The Chance to be Original and Creative
7. The Ability to Live and Work in the World of Ideas
8. The Hours and Security of the Job
9. Increased Autonomy within the Work

Which one or two do you believe are most important to technicians?

**MESSAGING**

If you had a technician who was more motivated by intrinsic values, how would you attempt to convince your technician to take on [task selected from study 1]?

If you had a technician who was more motivated by extrinsic values, how would you attempt to convince your technician to take on [task selected from study 1]?

**WRAP-UP**
Those are all my set questions. Do you have any additional information you would like to provide to the researchers regarding this subject?

Thank you for your time.
